# The role of *Serendipita indica* and *Lactobacilli* mixtures on mitigating mycotoxins and heavy metals’ risks of contaminated sewage sludge and its composts

**DOI:** 10.1038/s41598-020-71917-8

**Published:** 2020-09-16

**Authors:** Nesrine H. Youssef, Asma A. Al-Huqail, Hayssam M. Ali, Nader R. Abdelsalam, Mayada A. Sabra

**Affiliations:** 1 Regional Center for Food and Feed, Agricultural Research Center, Alexandria, Egypt; 2 Chair of Climate Change, Environmental Development and Vegetation Cover, Botany and Microbiology Department, College of Science, King Saud University, P.O. Box 2455, Riyadh, 11451 Saudi Arabia; 3 Timber Trees Research Department, Sabahia Horticulture Research Station, Horticulture Research Institute, Agriculture Research Center, Alexandria, 21526 Egypt; 4 Agricultural Botany Department, Faculty of Agriculture (Saba Basha), Alexandria University, Alexandria, 21531 Egypt

**Keywords:** Biological techniques, Genetics, Environmental sciences, Natural hazards, Pathogenesis

## Abstract

Accumulation of the Municipal Sewage Sludge (MSS) is considered as one of the most harmful renewable ecological and human health problems. MSS is a renewable resource that could be used as a soil organic amendment. This study aims to reduce the Heavy Metals (HMs) from the sludge content and sludge compost. Furthermore, this study is considered the first to assess the mycotoxins content in sludge and sludge compost via a new biological treatment using the fungus *Serendipita indica* or a mixture of lactic acid bacteria, thus providing safer nutrients for the soil amendment for a longer time and preserving human health. The HMs and mycotoxins were determined. The results exhibited that the biotic remediation of bio-solid waste and sewage sludge compost succeeded; a new bio-treated compost with a very low content of heavy metals and almost mycotoxins-free contents was availed. Also, the results indicated that the *Lactobacilli* mixture realized the best results in reducing heavy metals contents and mycotoxins. Afterward, *S. indica*. biotic remediation of bio-solid waste and sewage sludge compost minimized the health risk hazards affecting the human food chain, allowing for the different uses of sludge to be safer for the environment.

## Introduction

Despite the technological advance following the beginning of the nineteenth Century, the Egyptian farmers have utilized dried Municipal Sewage Sludge (MSS) treated with sulfuric acid or gypsum as organic fertilizer, commercially known as “Poudratte fertilizer”. This organic fertilizer is considered an excellent cheap bio-fertilizer because of the amount of organic matter and macro- and micronutrients in it^1,2^. Nowadays, as a result of the continuous increase of population and urban development, the sewage sludge production in Egypt is rapidly increasing; therefore, sludge needs to be managed to minimize the negative impacts of its application or disposal^3–5^. Sewage sludge disposal methods represent a critical environmental issue in Egypt. Recently, increasing concerns about sewage sludge management stemming from environmental risks surfaced, caused by the fast development of wastewater treatment plants without equal attention to treating the produced sludge. Therefore, there had been many attempts to reuse or manage this dried or dewatered sewage sludge for producing biogas and biofuel to generate electricity^6–8^. All these processes cause harmful emissions polluting the environment and contaminating the cultivated crops with airborne Heavy Metals (HMs) pollutants such as Ag, Al, As, Cd, Co, Cr, Cu, Fe, Hg, Mn, Mo, Ni, Pb, Sb, Se, Sm, Sn, Ti, V, and Zn which linger in atmosphere^9,10^ and different organs of plants^11^; thus, these components enter the food chain of humans, threatening their health due to their toxicity, even though it varies to degrees^12,13^ Unfortunately, although the government laws concerning the control and restriction of MSS and usage of sludge composts in planting wooden trees and ornamental plants, minimizing the accumulation of toxic pollutants and contaminants in the soil as well as its introduction to the food chain through crop plants in those lands is challenging. It also poses a risk to groundwater, surface water, and water bodies as a result of the runoff of pollutants. This type of soil pollution is not exclusive to developing countries as it was largely spread worldwide affecting many countries, such as France^14^, United States of America^15^, Canada^16^, United Kingdom, Sweden, Finland and Denmark (with acceptable limits for land application)^17^, Japan, Singapore, China, and Hong Kong^18^. The regulation laws of MSS or sludge recycling in agricultural usage are limited to 39.6% in 2015 and 35% until 2016 and 65% for fertilizers from the ashes of sewage-sludge incineration in the European Union^19^. Many efforts are made to minimize MSS fertilizer contents from biotic and abiotic pollutants such as bacteria, viruses, worms, fungi and dioxins, pharmaceutical drug traces, and heavy metals. Few studies were conducted about mycotoxins contamination^19,20^. Majeti et al.^21^ studied the removal of toxic metals such as copper, lead, cadmium, chromium, arsenic, and mycotoxins (aflatoxin B_1_, B_2_, M_1_, M_2_, G_1_, G_2_, patulin, ochratoxin A, deoxynivalenol, fumonisin B_1_ & B_2_, 3-acetyldeoxynivalenol, deoxynivalenol, fusarenon, nivalenol, diacetoxyscirpenol, HT-2, and T-2 toxin, and zearalenone and its derivatives) by using microbial biomass. This method was introduced as a cheap and novel promising method unlike conventional methods for the decontamination of food and raw materials^22^. Many attempts were applied to use the filamentous fungi like A.nidulans to degrade pollutants in wastewater^23^. Bacteria and fungi are the driving agents of composting^24^.

Lactic acid bacteria are mostly Gram-positive and can be classified as probiotics. The lactic acid bacteria are maintained for the most vital genera in the order *Lactobacilli* including *Lactobacillus*, *Leuconostoc*, *Pediococcus*, *Lactococcus*, *Streptococcus*, *Enterococcus*, *Weissela*, and *Bifidobacterium*. Recently, lactic acid bacteria and *S. indica* were used to treat the initial raw MSS to be almost safe to use and to be used later as biofuel to produce compost. Heavy metals can have disastrous effects on any living organism; probiotics could reduce metal toxicity as an alternative to conventional remediation methods^22^. *Lactobacillus* (*LAB*) *delbrueckii,* especially the subspecies (subsp.) *bulgaricus* KLDS1.0207, has a remarkable Pb binding capacity and the capacity of removing arsenic, cadmium lead, and silver^25,26^. Moreover, *Lactobacillus* spp., such as *L. casei* KC-324, inhibits the growth of *A. flavus* and reduces the produced aflatoxin^27^. *Lactobacillus* species mixture are more effective in reducing aflatoxin B_1_ than lactobacillus monoculture^28^*. S. indica,* formerly known as *Piriformospora indica*, is a root endophytic fungus with plant-promoting properties in numerous plant species that induces nutrient uptake and stress tolerance against abiotic and biotic stresses^29,30^. Also^31^, reported that several heavy metals, such as copper, were reduced by *S. indica* in sweet basil plants. Composting is a natural effective way to convert organic wastes into fertilizers and accelerate the reduction of certain toxic contaminants in sewage sludge^32,33^. The novelty of this study stems from the fact that is the first time to use this fungus and/or a mixture of two *LAB* bacteria as bio-removal agents for heavy metals and mycotoxins in MSS; we aimed to reduce the harmful effects of MSS compost, which is used in agriculture, to minimize its harmful effects on soils and to safely modify its use as a biofuel in a biotic manner to prevent adverse effects on the environment, underground water, air, vegetation, animals, and humans. Our choice of these bacteria mixture and fungus was based on their known efficacies according to literature, our pilot experiment, and their beneficial effects to soil and plants.

## Materials and methods

### MSS samples

Samples of seven days yield of raw Municipal Sewage Sludge (MSS) were randomly collected from seven points of the precipitated MSS basins at West Alexandria Sewage Sludge Treatment Station (Borgue El Arab) (30°50′56″ N 29°36′42″ E). Samples were uniformly mixed throughout the matrix in clean sterilized plastic containers. Air-dried fine powder of the MSS samples was gently homogenized and divided by a divider apparatus into subsamples, until the final result was four samples of 4.5 kgs of weight each appropriated according to the Investigative Operation Manual (IOM) of FDA, the part which contains the information on sampling details and sampling schedules for various occasions^34^. The moisture content of the air-dried raw MSS was measured using a Motomco apparatus serial no.K26242, USA. Then the moisture percentage was determined according to the Motomco index as 16% moisture at 23ͦC.

### *Lactobacilli (LAB)* mixture

*Lactobacilli* (*LAB*) capsules were brought from Ramida Egypt pharma, Tenth of Ramadan City, Egypt, under the license of Aptalis Pharma, France. Each capsule contains 2.5 billion CFU of *lactobacillus delbruckii* and 2.5 billion CFU of *Lactobacillus fermentum*.

### *Serendipita indica*

**Figure 1:**
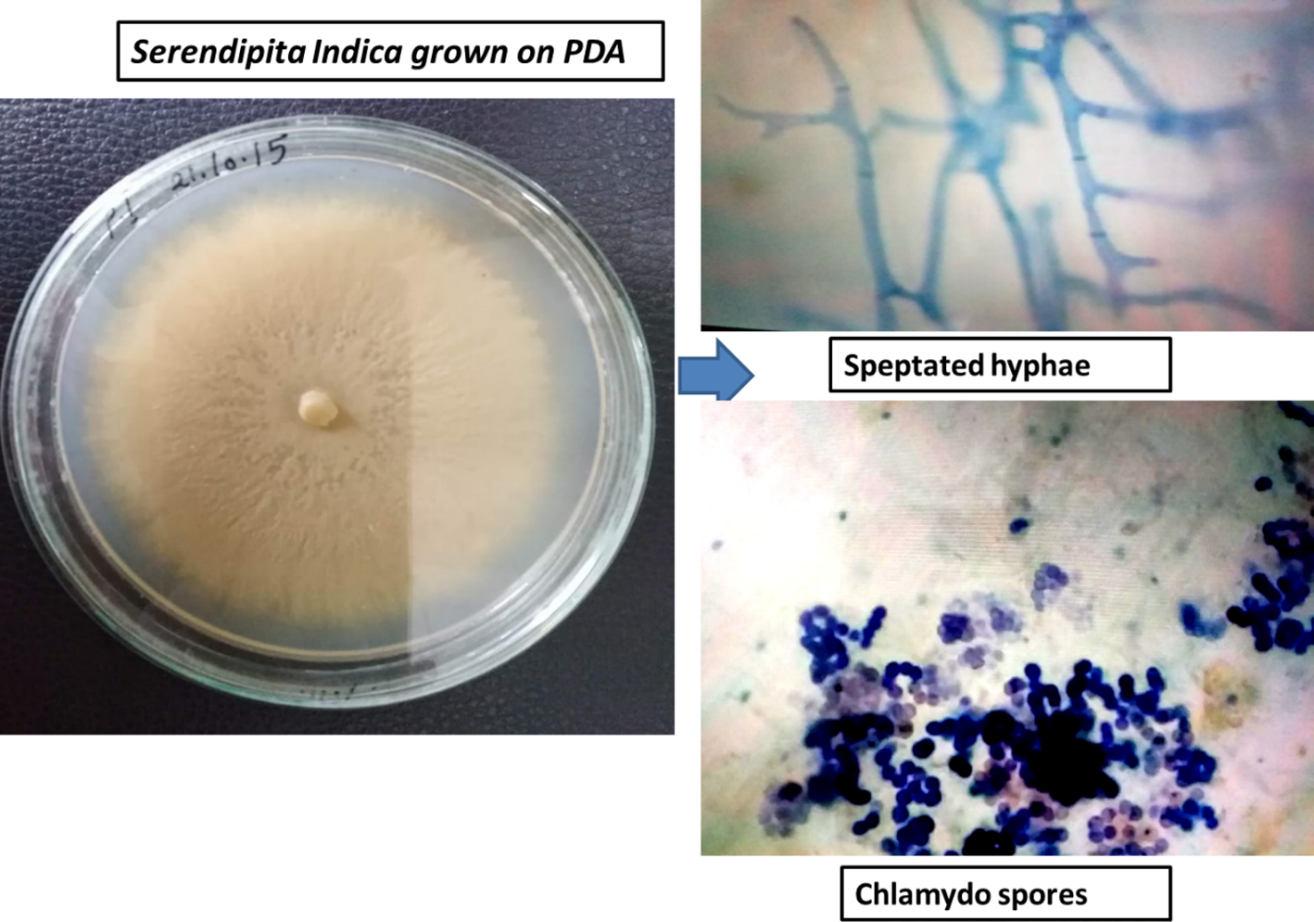
Morphological description of the used fungal strain *S. indica.*

*Serendipita indica* (strain DSM11827, NCBI tax. ID: 1109443) was grown on Potato Dextrose Ager media (PDA) plates for two weeks. A small hyphal plug was transferred into 500 mL conical flasks with liquid complete media 50 mL sterilized deionized water × 20 ml salt solution (120 g NaNO_3_, 10.4 g KCl, 10.4 g MgSO_4_ × 7 H_2_O, and 30 g KH_2_PO_4_ in 1 L), 20 g glucose, 2 g peptone, 1 g yeast extract, 1 g casamino acids, and 1 ml microelements (6 g MnCl_2_, 1.5 g H_3_BO_3_, 2.65 g ZnSO_4_ × 7 H_2_O, 750 mg KI, 2.4 mg NaMO_4_ × 2 H_2_O, 130 mg CuSO_4_ × 5 ml H_2_O dissolved in 1 L) which were incubated for three weeks in the dark at a temperature of 24^◦^C. The fungal strain is illustrated in Fig. 1.

### Clay soil

Clay soil was sterilized with 1% formalin. The soil was covered with a silver paper for one month until the formalin traces disappeared entirely. Then, the soil was kept in a sterilized bag until its usage.

### Rice straw

Solar-dried rice straw was sterilized with sodium hypochlorite 1% v/v and oven dried at a temperature of 45 °C for 24 h. Then, the rice straw was kept in a sterilized bag until its usage.

### Preparation of samples for mycotoxins detection

Representative homogenized subsamples were taken from six points of the four tested samples.

#### Determining the mycotoxins content

Preliminary mycotoxins are qualitatively detected using Thin-Layer Chromatography (TLC) according to Gilbert and Anklam^35^. Then, mycotoxins are quantitatively detected using a detection method in soil according to Uhlig et al.^36^ with certain modifications. MSS samples, 20 g each, were extracted with 100 ml methanol–water (9:1). Then, the samples were blinded then centrifugated at 3,000 RPM, and each supernatant was purified using Whatman filter paper (40 Millipore’s). Mycotoxins occurrence in the samples were determined using TLC plates and two eluents TEF (toluene/ethylacetate /90% formic acid 5:4:1.v/v/v) and TAM (toluene/acetone/methanol 5:3:2, v/v/v) according to^37^ was applied. The developer Anis (0.5% p-anisaldehyde in methanol/acetic acid/conc) and sulfuric acid (17:2:1 v/v/v) was heated for eight minutes at a temperature of 130 °C. The dry-developed TLC plates were viewed under longwave (366 nm) and shortwave (254 nm) UV light and compared with the published data on color and Rfg values (Retardation Factors relative to Griseofulvin for some known mycotoxins^37^. The present mycotoxins types are registered. After determining the type of mycotoxins, A phenyl hexyl column was used to separate each mycotoxin in the final MSS extract according to to^38^ with certain modifications. Each mycotoxin was analyzed using High-Pressure Liquid Chromatography (HPLC) with fluorescence detection. Mycotoxin standards are brought from Sigma Aldrich, Cairo bureau.

#### Confirmatory test

To eliminate other undesired co-extracted matters from MSS, the hair bait analysis technique was applied according to Jogawat et al. and Nosratabadi et al.^29,39^ with further development. The pH values of MSS were measured according to^40^. The removal of horse-hair fatty traces was done by soaking them for 24 h in diethyl ether and rinsing them for 4–5 times with distilled water followed by air drying. Keratinophilic fungi were isolated by the hair-baiting technique where horsehair was used as a keratin bait source. 12 sterile Petri dishes were half filled with MSS and three pieces of marked horsehairs tips with colored sterilized stickers which were 2–3 cm each. The stickers were spread over the surface of MSS. The MSS samples were moistened with sterile distilled water and then were baited by burying sterile marked horsehair pieces in the MSS. These dishes were incubated at a temperature of 30 °C for 15 days then the horsehair pieces were gently removed to inoculate three potato dextrose broth conical flasks, which were 100 ml each, supplemented with streptomycin (30 mg/l.) and then incubated at a temperature of 30 °C for 7 days. At the end of the incubation period, the same phenyl hexyl column was used and each mycotoxin was detected using the HPLC apparatus with fluorescence detection according to^38^.

#### Screening of total heavy metal elements in the raw MSS samples

Heavy metals were determined in the representative samples using the analytical device ICP-ES Agilant 5100VDV of Inductively Coupled Plasma Atomic Emission Spectroscopy (ICPOES), an analytical technique used for the analysis of trace metals with high accuracy and precision and low detection limit starting at ppb levels to the high concentration of ppm. The metals’ analysis process and quality control were carried out according to^41,42^**.** The process occurred in the ICPOES *LAB*, Institute of Graduate Studies and Research, Alexandria University, according to the standard methods of US EPA (2007). All the detected elements (Al, As, Cd, Cu, Fe, Pb, and Zn) have been accredited according to ISO 17,025.

### Biotic treatment of raw municipal sewage sludge

#### The effects of the biotic treatment in reducing mycotoxins contents and heavy metals (HMs) concentration levels in raw MSS

##### Testing the activity of *Serendipita indica* in low and high pH solutions

The optimal pH for growing *S. indica* to obtain the higher fungal growth mat mass was tested to assess the extent of the enhanced probability of reducing mycotoxins and removing HMs. The experiment was carried out by growing *S. indica* on Complete Media (CM) 50 ml under many acidic and basic pH values at 4, 5, 7.8, and 8.2, successively, for 15 days at a temperature of 25 °C. At the end of the incubation period, the fungal growth mat mass was dewatered and weighed. Then, the weight of *S. indica* growth mass was registered as showed in Table 1. After determining the optimal pH for *S. indica* growth. The Complete Media (CM), 150 ml each, was adjusted at the determined pH then was inoculated with *S. indica* and incubated at a temperature of 25 °C for 15 days.

##### The effects of *Serendipita indica* in reducing mycotoxins contents and heavy metals (HMs) concentration levels in raw MSS

Six cylinders, 2000 ml each, were used in this experiment. Each cup contained 300 g MSS, 1,350 ml deionized sterilized water, and 150 ml medium of *S. indica* growing on potato dextrose medium broth under the determined pH value. These parameters were utilized to reach a final liquid volume of 1,500 ml. After that, each flask was covered with a plastic cover 3 cm in depth, then incubated for 15 days at a temperature of 25 °C to simulate the real situation in the treatment process a covered tank is used; each tank is 100 kg each. Control samples, three cylindrical cups 1,000 ml each, were used in all the experiments, which consisted of 100 g MSS + 350 ml distilled deionized water or/and 150 ml potato dextrose broth adjusted to a pH value of 4. At the end of the incubation period, the fungal growth mat mass growth was gently removed. The excess water was disposed of, and then the treated sewage sludge silt was obtained. The mycotoxins were detected using the HPLC apparatus with fluorescence detection according to^38^ and the heavy contents were gauged according to^41,42^.

##### The effects of *LAB* mixture in reducing mycotoxins contents and heavy metals (HMs) concentration levels in raw MSS

Only acidic pH is used in this experiment because *the LAB* is known to be more active and effective in an acidic pH value of 5 according to literature^43^. The *LAB* mixture content of one capsule (2.01 g which form 5 billion CFU) was suspended in sterilized autoclaved Bacto B12 culture broth 150 ml each; their parameters are recommended for maintaining the stock culture of LAB according to U.S standards with certain modifications. Then, incubation occurred at a pH value of 4.8 and a temperature of 25 °C for 24 h; 25 °C was the suitable temperature for *S.indica* growth^31^, and *LAB* metabolites have higher antiaflatoxigenic activity at a temperature of 25 °C^27^. Meanwhile, at a temperature of 25 °C, the production of the mycotoxin was the highest^44^. Three cylinders, 2000 ml each, were used in this experiment. Each containing 300 g MSS, 1,350 ml deionized sterilized water, and 150 ml of the LAB mixture medium. The same steps, as mentioned in the *S. indica* experiment, were repeated. At the end of the 15^th^ day, the water excess was disposed, and the treated sewage sludge silt was obtained. The mycotoxins were detected using the HPLC apparatus with fluorescence detection according to Zhang et al.^38^ and the heavy contents were determined according to Ilieva et al. and Tytła^41,42^. The resulting data are registered and compared with the *S. indica* results.

### Composting processes of MSS

This experiment’s main target was to minimize the mycotoxins and heavy metals contents in Municipal Sewage Sludge (MSS) to reduce the pollution of soils, groundwater, and plants and prevent them from reaching the human food chain. The composting processes of MSS were carried out under aerobic conditions using three composting methods as follows:

#### The roles of conventional MSS compost

Pretreated MSS compost, and biotic MSS compost in reducing mycotoxins contents and heavy metals concentration.

#### Preparation of rice straw and clayey soil samples

Samples of rice straw and clayey soil were taken for mycotoxins estimation and heavy metals determination according to Ilieva et al. and Zhang et al.^38,41^. Then, the samples were surface sterilized using sodium hypochlorite (75%) and washed with sterilized deionized water three times and oven dried for one hour at a temperature of 45 °C after getting rid of excess water.

### The conventional composting processes

The conventional composting process in the field was mimicries under in vitro conditions but within a small scale^45^. The Dried Municipal Sewage Sludge (DMSS) was taken then distributed into a pile. Each pile (40 cm in height × 75 cm in length and 2.25 to 2. 50 kg in weight) contained DMSS covered with a clay soil layer then a rice straw layer was added then another DMSS layer. The process was repeated until getting a large pile or heap as recommended according to^46^ was formed. The compost was incubated under aeration conditions at a temperature of 65 °C for 50 days according to Lu et al.^46,47^. At the end of the incubation period, a representative sample was taken for mycotoxins estimation and heavy metals determination according to Ilieva et al. and Zhang et al.^38,41^**.**

#### The composition process with pretreated MSS

The modified composition process was carried out using pretreated DMSS with *S. indica* and/or *LAB* mixture under the same in vitro conditions with the same steps of the conventional composition process as mentioned above. After 50 days, a representative sample was taken for mycotoxins detection and heavy metals determination as mentioned above.

#### The biotic composition processes

The biotic composition process was carried out using pretreated DMSS with *S. indica* and/or *LAB* mixture with the addition of *S. indica* and/or *LAB* mixture cultures as follows: pieces of sterile gauze (10 × 20 cm each) were planted separately in 500 ml broth culture of each of the *LAB* mixture and/or *S. indica* and incubated for seven days at a temperature of 25 °C. At the end of the incubation period, the inoculated gauze was gently removed and placed between layers of the compost pile as an additional layer with a ratio of 1:6 layers under the same in vitro conditions with the same steps in both conventional composition and pretreated methods. After 50 days of incubation under aeration conditions, according to Stegenta et al.^5^ the gauze was removed and representative samples were taken for mycotoxins detection and heavy metals determination as mentioned above.

### Statistical analysis

The first experiment was carried out in three replications in a completely randomized design. Meanwhile, the second experiment was carried out in three versions in a randomized complete-blocks design. The data were subjected to statistical analysis using a Costat computer package (CoHort Software, Berkeley, CA, USA). One-way completely randomized and/or randomized complete blocks ANOVA were used and a comparison between the resulting data was done using Least Significant Difference (LSD) and the correlation between the tested treatment and the removal process was calculated according to Duncan’s multiple range test, which was applied to compare the treatment mean values according to^48^.

### Ethical approval

“This article does not contain any studies with human participants performed by any of the authors”.

## Results

The current results will be divided into two partitions as follows:

### Experiment I: assessment of mycotoxins contents and total heavy metals concentration in raw municipal sludge and testing the effects of biotic treatments on reducing their contents

#### The effect of the medium’s pH value on the growth of *S. indica*

**Figure 2:**
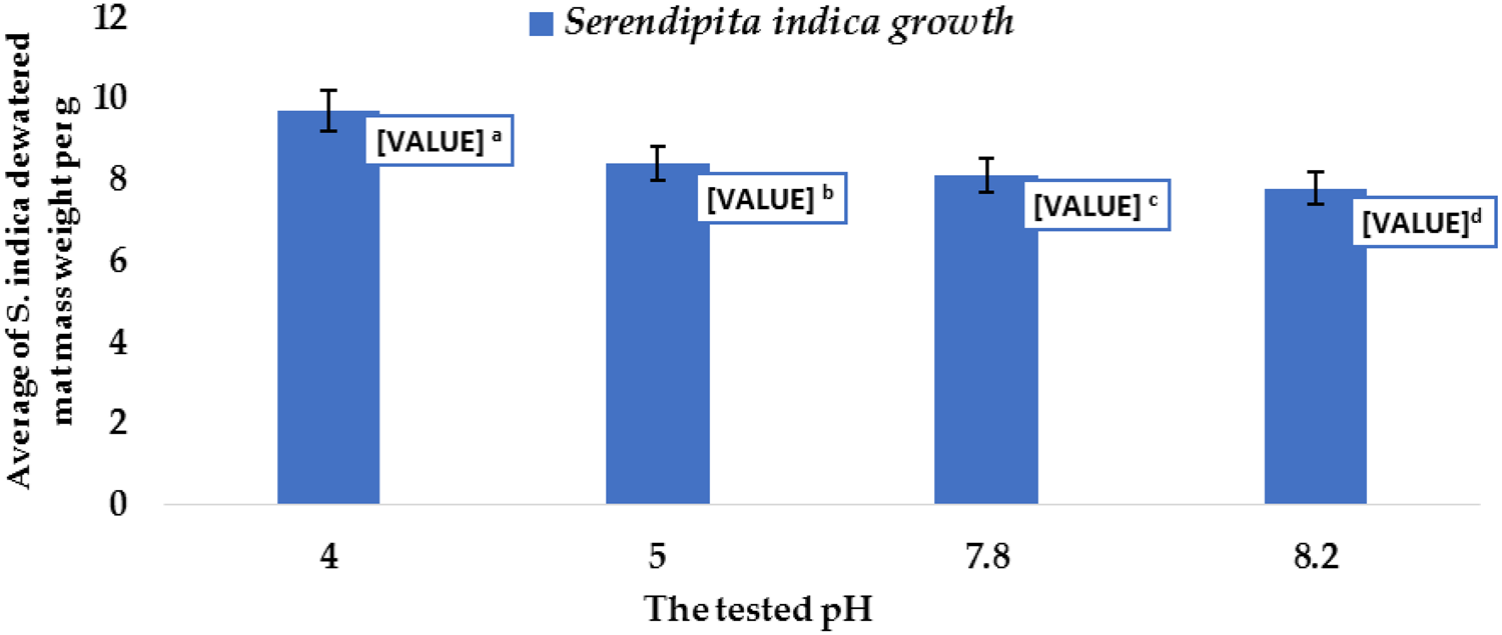
Effect of different pH numbers on *S. indica* growth.

During the current study, different pH values were tested to study their effect on the growth of *S. indica*. The results in Table 1 and Fig. 2 illustrate that the difference in pH values significantly affected the *S. indica* growth and pH value 4 achieved the highest fungal growth mat mass (9.72 g), while pH value 8.2 recorded the lowest value which was 7.81 (g) with L.S.D._0.05_ = 1.13. So, the growth medium of *S. indica,* which will be used in all the experiments of this current study, will be adjusted to pH value 4.

**Table 1:** Effect of biotic treatments on mycotoxin contents in sludge.

Mycotoxins	Raw sludge (control)	PDAmedium + sludge (pH = 7.82)	Acidified PDA medium + sludge (pH = 4.0)	*Serendipita indica* + sludge (pH = 7.82)	*Serendipita indica* (growing at + sludge (pH = 4.0)	*Lactobacillus* Mixture + sludge (pH = 4.8)	LDS0.05
		Incubation for fifteen days at 25 °C					
Aflatoxin B_1_ (ppb)	69^b^	72^a^	67.0^c^	16.80^d^	6.240^e^	6.93^e^	1.781
Viomelline(ppb)	3400^c^	5790^a^	5600^b^	3,093.75^d^	219.0f.	293.34^e^	8.3445

Treatments with the same letters are not significantly different. where the pearson Product Moment Correlation coefficient of the two mycotoxins and the inhibition process are significant were S.E of r 0.103593for aflatoxin and 0.1177833for viomellin and p(r = 0) 0.000*** for both mycotoxins.

#### Estimation of mycotoxins contents in raw municipal sludge

**Figure 3:**
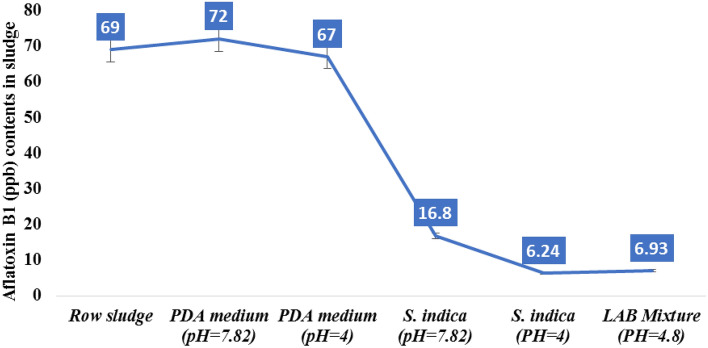
Effect of biotic treatments on Aflatoxin B1 (ppb) contents in sludge.

The results in Table 1 and Fig. 3 illustrate the following:

The occurrence of two mycotoxins imposes the probability of being produced by more than one fungus. So, the action of *S. indica* and *LAB* mixture against these fungi was different. Moreover, the increase in *afLAB*_*1*_ and *viomelline* concentrations during the incubation in the PDA medium indicated that the natural biotic flora of MSS is still active after all the treatment steps such as coagulation treatments with polyacrylamide. Furthermore, in the case of aflatoxin B_1_, the reduction of *afLAB*_*1*_ amounts by both *S. indica* (in acidic pH) and/or* LAB* mixture was not significantly different. The results in Table 1 indicated that *S. indica* was more effective in reducing viomelline than LAB mixture which indicated that the capability of the fungus in reducing mycotoxins varied according to the mycotoxin type. The sensitivity of fungus-producing mycotoxins to *S. indica* antibiosis and *LAB*’s enzyme system was evident.

According to results as illustrated in Table 1 and Fig. 3, the aflatoxin B_1_ was 69 part per billion (ppb) and viomelline was 3,400 ppb in raw sludge (the control group), with the selected pH value 4 as pointed in Fig. 3. Using *S. indica* decreased the *Aflatoxin B*_*1*_ to 6.240 ppb compared with 6.93 ppb under LAB mixture with no significant variations between both treatments. Also, these results detected that *S. indica* decreased the viomelline (219 ppb) more than the LAB mixture which was 293.34 ppb as recorded in Table 1. While under the high pH value 7.82, the results showed a decrease in *Aflatoxin B*_*1*_ which was 16.80 ppb, but this value occurred more than two instances under pH value 4 by using *S. indica.* That proved the results in Fig. 1. Our results illustrated that this primary treatment of the raw MMs with both fungus and LAB mixture keeps the raw sludge almost safe because these concentrations are near the permissible level limits of aflatoxin B_1_ (5 ppb) in most of countries^49^.

#### The effects of biotic treatments on heavy metals concentration in raw sludge

**Figure 4:**
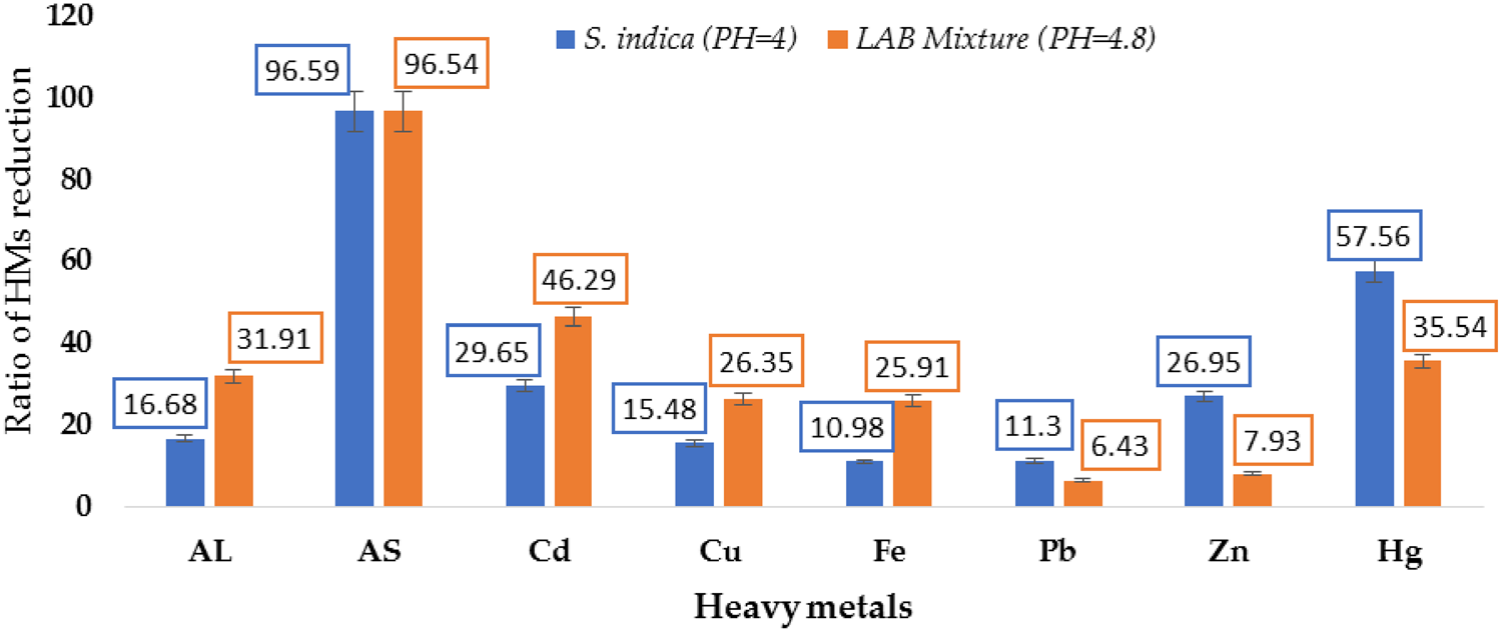
Efficacy ratio of heavy metal concentrations reduction treated with *S. indica* (pH = 4) and Lactobacillus mixture (pH = 5.5).

The results are exhibited in Table 2 and Fig. 4 illustrated that the effectiveness of each treatment in removing heavy metal (HMs) elements varied according to the element type. Furthermore, in the case of *S. indica*, pH = 4 was more effective in reducing the HMs concentration except for cadmium which showed the lowest value at 2.41 compared with the control value 3.43. The removal of arsenic (As) was higher than mercury Hg > Cd > Zn > Al > Cu > Pb > Fe. An efficacy percentage of 96.59% was evident for heavy metal concentrations reduction. Whereas, at basic pH values, the *S. indica* efficiency for removing or digesting heavy metals was as the following order: As > Hg > Cd > Al > Zn > Pb > Fe > Cu. These heavy metals have an atomic density higher than 4 g/cm^3^, and these include lead (Pb), cadmium (Cd), zinc (Zn), mercury (Hg), arsenic (As), copper, and Iron. The plant absorption of these minerals and their accumulation in food crops are damaging human health.

**Table 2:** Effect of biotic treatments on heavy metals contents in raw sludge (mg/L).

Heavy metal concentration in ppm after incubation for fifteen days at 25 °C							
	Raw sludge (control)	Incubated sludge on PDA at pH = 7.8	Incubated sludge on acidic PDA	Sludge treated with *S. indica* at pH = 7.82	treated with *S. indica* at pH = 4	Sludge treated with *Lactobacillus* mixture at pH = 5.5	L.S.D_0.05_
		Incubation at 25 °C for fifteen days					
AL	15,446.85^a^	15,145.32^b^	14,491.12^c^	13,903.70^d^	12,869.73^e^	10,517.79f.	0.14670.141
HMDER%	–	1.95	6.18	9.99	16.68	31.91	–
AS	567.19^a^	208.87^b^	42.52^c^	19.84^d^	19.33f.	19.65^e^	0.052470.005
HMDER%	–	63.17	92.51	96.50	96.59	96.54	–
Cd	3.43^a^	2.10 ^c^	2.45^b^	2.02 ^d^	2.41 ^b^	1.84f.	0.04040.04044
HMDER%	–	38.64	38.64	41.22	29.65	46.29	–
Cu	305.38^a^	293.89^b^	293.15 ^c^	281.84 ^d^	258.08 ^e^	224.89 f.	0.05240.05237
HMDER%	–	3.76	4.00	7.70	15.48	26.35	–
Fe	25,256.62^a^	23,600.06^bbbbbbb^	22,968.37^c^	22,573.78^d^	22,481.16^e^	18,713.30f.	0.1880.14888
HMDER%	–	6.56	9.06	10.62	10.98	25.91	–
Pb	235.78^a^	231.93^b^	213.28 ^d^	209.98^e^	209.14f.	220.62 ^c^	0.04990
HMDER%	–	1.63	9.54	10.94	11.30	6.43	–
Zn	1,062.21^a^	1,042.74^b^	993.93^c^	933.400 ^e^	775.93f.	978.25 ^d^	0.03630
HMDER%	–	1.83	6.43	12.15	26.95	7.93	–
Hg	750.40^a^	726.36^b^	699.76^c^	407.5^e^	318.5f.	483.75^d^	0.2075.002
HMDER%	–	3.20	6.75	45.69	57.56	35.54	–

Element	S.E of r	P(r = 0)					
Al	0.11109849	0.0000***					
As	0.1458394	0.0000***					
Fe	0.1088228	0.0000***					
Cu	0.11066159	0.0000***					
Cd	0.1275773	0.000***					
Pb	0.18132387	0.0016**					
Zn	0.1876714	0.0027**					
Hg	0.1409849	0. 0000***					

Where, HMDER% = heavy metal degradation efficacy reduction. Reduction. One way Anova completely Randomized with probability; p*** where the pearson Product Moment Correlation coefficient of the heavy metal elements are exhibited in the table below.

In the case of the *LAB* mixture. This treatment realized the highest efficacy ratio in removing Al, Cd, Cu, and Fe in comparison with *S. indica*. The efficacy of the *LAB* mixture and *S. indica* in removing arsenic was the highest. Unfortunately, despite the success of both the *LAB* mixture and *S. indica* in reducing concentrations of heavy metals in raw sludge, the concentrations of these heavy metals, except Cd and Pb, are still high compared to the permissible levels whether in air^50^, plants, or soil. On the other hand, for the treated MSS which could be added to soil to fertilize woody trees, the biological treatment of MSS with *S. indica* was more effective in reducing arsenic (As) than the *LAB* mixture. Meanwhile, the *LAB* mixture was only effective in reducing Al to be in the permissible level of HMs in soil (10,000–300,000 ppm). Both biotic treatments reduce Cd and Pb concentrations to be within the allowable limits of the European Union standards (3 and 300, respectively).

### EXPERIMENT II: the effects of composting processes of raw municipal sewage sludge on mycotoxins and total heavy metals contents

Mycotoxins were determined quantitatively using HPLC and heavy metals were determined using ICPOES as stated in Table 3. It was shown that the mycotoxins contents (ppb) were *viomelline* (4,903.78 ppb), *aflatoxins* (20.90), and *moniliformin* (2,688) in rice straw, while *viomelline* and *moniliformin* were 0.0 in clay soil and *aflatoxins* was 2.293 ppb. Also, the data in Table 3 recorded the element concentrations. The results in Table 4 exhibited that the conventional process can reduce MSS mycotoxins content by 36.23%, but it was more effective in reducing *viomelline* than aflatoxin by 79.45%, which indicated that aflatoxin may be more heat stable than *viomelline*.

**Table 3:** Determination of mycotoxins and total heavy metals contents in compost components (rice straw and clay soil).

Materials	Mycotoxins contents (ppb)			Element concentration (ppm)							
	Viomelline	Aflatoxins	Moniliformin	Cd	Cu	Fe	Al	Pb	Zn	As	Hg
Rice straw	4,903.78	20.900	2,688.0	0.073	26.803	452.87	326.16	5.22	54.60	5.76	0.0
Clay soil	**0.0**	2.293	**0.0**	3.525	5.493	7,998.44	4,595	11.78	37.22	0.0	0.0

**Table 4:** Comparison between raw Mss and conventional MSS composting method on global mycotoxins contents and heavy metal concentrations.

Treatment	Aflatoxins	Aflatoxin inhibition.ER%	Viomelline	Viomelline inhibition ER%	Moniliformin	Moniliformin inhibition ER%
Raw MSS	69^a^	–	3400^a^	–	0.0^b^	–
Conventional compost	44.00^b^	36.23	698.63^b^	79.45	388.8^a^	85.536
L.S.D._0.05_	4.534		3.206		0.1603	

Where, ER% = efficacy ration of mycotoxin inhibition process, treatments with different letters are significantly differentData were analyzed as Anova one way completely randomized design with p*** where the pearson Product Moment Correlation coefficient of the three mycotoxins and the inhibition process are significant were S.E of r 0.124486 for aflatoxin ,0.02773 for viomellin and 0.07644 0.07644 for moniliformin and p(r = 0) 0.000***,).0.001***and 0.0000*** successively.

The results, as exhibited in Table 5, illustrated that the primary biotic treatment before the composting process with probiotics or/and *S. indica* reduced the concentrations of the occurred mycotoxins more than the conventional compost. *S. indica* was more effective in reducing the three occurred mycotoxins more than the *LAB* mixture. The efficacy ratio of mycotoxins reduction was higher in aflatoxin followed by *viomelline* then moniliformin. The biotic composting process realized the higher mycotoxins reduction percentages in comparison with the other composition processes. The composting process with *S. indica* eliminated aflatoxins followed by moniliformin then *viomelline,* whereas *LAB* was the best in completely removing *viomelline* followed by aflatoxins, and the biotic composition process was safer to be added to soil followed by pretreated composting process Our results coincide with those of who reported that *Lactobacill*i could remove aflatoxin B_1_ with a removal rate of 50–80%. But our treatments with the LAB mixture and/or *S.indica* are approximately similar to those of Huang et al. ratios (65.68 for LAB to 68.28 for *S.indica*) but the biotic composting process was more effective (99.26) in case of *LAB* to − 100% in case of *S.indica*. Furthermore, according to Gourama and Bullerman^28^, The mixture of *Lactobacilli* species not only inhibited aflatoxin production but also completely inhibited the germination of mold spores.

**Table 5:** Comparison between the effects of the three-composting process on mycotoxins reduction.

Conventional composting process				Pretreated composting process								Biotic composting process					
**Mycotoxins**																	
	Viomelline (ng/g)	Aflatoxins (ppb)	Moniliformin (ng/g)	Viomelline (ng/g)		Aflatoxins (ppb)			Moniliformin (ng/g)			Viomelline (ng/g)		Aflatoxins (ppb)		Moniliformin (ng/g)	
				M.C + *S. indica*	M.C + L.B	M.C + *S. indica*		M.C + L.B	M.C + *S. indica*	M.C + L.B		M.C + *S. indica*	M.C + L.B	M.C + *S. indica*	M.C + L.B	M.C + *S. indica*	M.C + L.B
Mycotoxin concentration	698.63^a^	44.0^a^	388.8a	289.5^cb^	326.64^b^	13.955^b^		15.02^b^	203.148^c^	228b		189.00^cdd^	0.0^ed^	0.0^c^	.c324^c^	50.209^e^	65.8^d^
ERMR %	–-	–-	–	58.562	53.25	68.28		65.86	47.75	41.36		72.95	100	100	99.26	77.97	71.14
L.S.D_.05_	0.83076	2.0623	0.829677	0.83076		2.0623			0.829677			0.83076		2.0623		0.829677	

Where, ERMR % = efficacy ratio of mycotoxin reduction, treatments with different letters are significantly different. Data are analyzed as Anova Randomized complete blocks design with highly significant, p*** where the pearson Product Moment Correlation coefficient of the three mycotoxins and the inhibition process are significant were S.E of r 0.0649895 for aflatoxin 0.064468 for viomellin and 0.064468 for moniliformin so P(r = 0) = 0.0001***.

**Figure 5:**
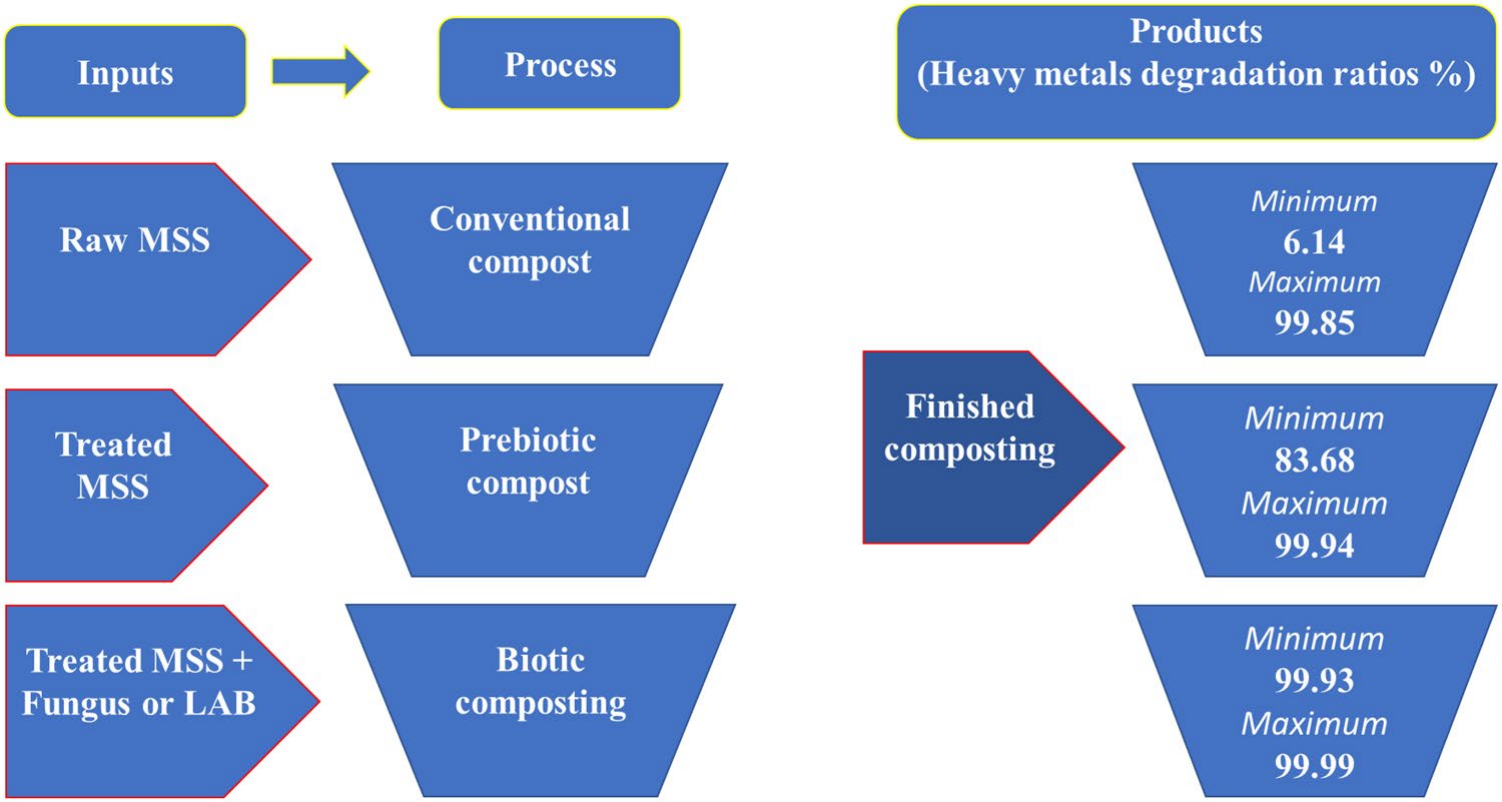
Effect of composting types on heavy metals degradation.

The data in Table 6 and Fig. 5 demonstrated the followings results: The composting process of MSS was vital to the minimization of the HMs concentrations more than raw MSS. The HMs removing effectiveness and efficiency occurred in this order: the biotic composting process > pretreated composting process > conventional composting process. Each composting process differed in their HMs–removing efficacy according to the removal elements. In the case of the pretreated composting process, the *LAB* mixture was more effective than *S. indica* when interacting with Al and Cd, whereas this effect was similar to *S. indica’s* considering As, Fe, and Cu. On the other hand, the removal efficiency of *S. indica* was higher in the case of Pb, Zn, and Hg. In the case of the biotic composting process, the LAB mixture was significantly effective in reducing Al, Fe, zinc, and Pb rather more than *S. indica’s* binding efficacy. Meanwhile, both of the *LAB* mixture and *S. indica* were similarly effective in reducing As, Cd, Cu, and Hg. All resulting heavy metal elements concentration, except Al, were below their normal range in soil and plant according to their permissible limits recommended by WHO, FAO, Dutch, Austrian, Russian, and European Union standards. These results, as illustrated in Tables 5 and 6 and Fig. 5, indicated also that the biotic compositing can provide nutrients for soil amendment safely. These findings were closely in agreement with those of^51^ who reported that the microbial addition during composting is considered highly effective due to the production enhancing of different enzymes resulting in rate acceleration of waste degradation. After 25 days, the biodegradation ability of these microbes was not affected after being added to the soil. Our results match^51^ who used *Aspergillus niger* to removing heavy metals via a bioleaching process. Even though the performance of their method was more effective in reducing Pb and Zn more than our treatments, our method was more effective in reducing Fe, Cu, Cd, As, and Al. Moreover, the bioleaching mechanism augments the metal solubilization which increases their mobilities and, consequently, their bioavailability. Alternatively, biological agents, such as *Phanerochates chrysosporium* and *S.indica* which have been used in removing Pb and Cu, respectively^31^, can accumulate the high concentration of heavy metals in the nontoxic forms and be capable of reducing their toxic effects by using them during their physiological metabolism.On the other hand, the LAB mode of action in removing heavy metals was based on H.M binding capacity^22^ which decreased their bioavailability.

**Table 6:** Comparison between the effects of the three-composting process on heavy metals (H.M) removing.

Elements (ppm)	Raw MSS	Conventional compost	Pretreated compost		Biotic compost		L.S. D_0.05_
			*S. indica*	*LAB* mixture	*S. indica*	LAB	
Al	15,146.84^a^	7,557.8^b^	2,520.7^c^	2,205.3^d^	3.59^e^	2.77f.	0.0426659. 96
HMDER %	–	51.07	83.68	85.72	99.97	99.98	
As	567.19^a^	17.92^b^	5.99^c^	5.99^c^	0.02^d^	0.023^d^	0.4913569. 32
HMDER %	–	96.83	98.95	98.94	99.99	99.99	
Fe	25,256.62^a^	21687^b^	3,616.2^c^	3,615.84^c^ c	7.98^d^	5.58^e^	0.737259
HMDER %	–	14.13	85.68	85.68	99.96	99.97	
Cu	305.37^a^	286.63^b^	47.82^c^	47.80^c^	0.19^d^	0.14 ^d^	0.053968. 2
HMDER%	–	6.139	84.37	84.35	99.93	99.95	
Cd-	3.43^a^	1.45^b^	0.36^c^	0.24^d^	> 0.0001e ee	> 0.0001e e	0.019082
HMDER%	–	57.54	89.36	92.88	99.99	99.99	
Pb-	235.78^a^	192.39^b^	64.20^c^	48.14^d^	0.15^e^	0.09 f.	0.039531
HMDER%	–	18.40	72.77	79.58	99.93	99.96	
Zn	1,062.21^a^	930.14^b^	232.66^d^	271.45^c^	0.51^e^	0.42 f.	0.038877.05
HMDER%	–	12.42	78.09	74.45	99.95	99.95	
Hg	750.40^a^	0.75^b^	0.49^c^	0.59^d^	0.2^e^	0.19^e^	0.044189.033986
HMDER%	–	99.85	99.94	99.89	99.97	99.97	

Element	S.E of r	P (r = 0)					
Al	0.10964	0.0001***					
As	0.184746	0.0022**					
Fe	0.1075639	0.0000***					
Cu	0.089057	0.0000***					
Cd	0.17809	0.012**					
Pb	0.082601	0.0000***					
Zn	0.09408	0.0000***					
Hg	0.18887	0.032**					

Where HMDER% = heavy metal degradation efficacy ratio. Data are analyzed as Anova Randomized complete blocks design with highly significant, p*** where the pearson Product Moment Correlation coefficient of the heavy metal elements are exhibited as a table below.

## Discussion

The usage of the *LAB* mixture *or S.indica* in removing mycotoxins not only minimized or inhibited the mycotoxins naturally contaminated Mss but also exhibited how their metabolites affect the molds’ cell wall. Meanwhile, the competition between *S. indica* and other filamentous fungi-contaminated Mss on carbon sources caused a sort of carbon starvation which induced autolysis, cell wall degradation biopolymers, and mycotoxin production. Moreover, in this study, we tested different pH values to study their effects on the growth of *S. indica*. The current results showed the difference in pH values significantly affected the *S. indica* growth and pH value 4 achieved the highest mass mat growth (9.72 g). These findings agreed with those of^31^. For the estimation of mycotoxins contents in raw municipal sludge, the results showed that *S. indica* and the LAB mixture against these fungi were not similar and the increase in *afLAB*_*1*_ and viomelline concentrations indicated that the natural biotic flora of MSS is still active after all the treatments applied in the treatment station. Furthermore, in the case of aflatoxin B1, the reduction of *af LAB*_*1*_ amounts by both *S. indica* (in acidic pH) and/or *LAB* mixture was not significantly different. These findings were matched with those of^52,53^ who found that fungal endophyte reduced trichothecenes and zeralenone in maize. Our results are also in harmony with those of^52,53^ who mentioned that lactic acid bacteria prevents the *Aspergillus flavus* growth and the produced mycotoxin because of their ability to bind with aflatoxins in aqueous solutions.

Our results indicated that *S. indica* was more effective in reducing viomelline than *LAB* mixture and these results are meaningful like those of^54^ which found that the *LAB* kefir binding *aflatoxinB*_*1*_ is more effective than *zearalenone* and *ochratoxin*. Our results also coincided with^55,56^ who mentioned that the *LAB fermentum, LAB gallinarum,* and *LAB reuetri* were only capable of binding *aflatoxinB*_*1*_ among seven isolates, and the *LAB plantarum* strains produced enzymes such as α- amylase, esterase, lipase, and α- glucosidase which can differ in their activities according to the fungus-producing mycotoxin. According to our study on the effect of biotic treatments on heavy metals concentration in raw sludge, the results showed the effectiveness of these treatments in removing Heavy Metal (HMs) elements varied according to the element type. *S. indica*, (pH = 4) was more effective in reducing the HMs concentration. In case of the *LAB* mixture, it realized the highest efficacy ratio in removing Al, Cd, Cu, and Fe in comparison with *S. indica*. On the other hand, for the treated MSS which could be added to soil to fertilize woody trees, the biological treatment of MSS with *S. indica* was more effective in reducing arsenic (As) than the *LAB* mixture. Meanwhile, the LAB mixture was only effective in reducing Al to be within the permissible level of HMs in soil (10,000–300,000 ppm as maintained by^57^. This finding was closely in agreement with^58^ who reported that that natural occurrence safe levels of Cd in Ethiopian soil were 2.3–2.5, whereas our resulted Cd concentration levels after biotic treatments were 1.8–2.4 and Pb were 209.9–220.6. Other HMs were higher than the permissible limits, which may occur due to the short period of incubation. This indicates that this biotic remediation process (after this applied incubation period) was not enough for removing all the HMs and for the safe addition of treated MMs to soil.

Our results exhibited that pH values have an important effect on HMs concentrations. This finding was generally in harmony with those of^57^ who reported that pH values significantly affect the solubility and mobility of As, Cr, Pb Cu, Ni, and Zn metals. Our data matched those of^59^ who mentioned that Hg was adsorbed at low pH values more than high pH values. On the other hand, the determined heavy metals in raw MSS, which can be used as biofuel or to generate electricity, were present in very high concentrations. This indicates that this treatment of Mss was not enough to mitigate the HM risks for human health and air pollution. Our recorded data matched those of^60^ who mentioned that air pollution is caused by the high levels of heavy metals in the atmosphere. Also, the values matched with those of the permissible levels of the European Commission. Furthermore, the permissible level of each heavy metal intake and uptake via respiratory, exposure, and dietary routes of humans differed between countries^61^. To study the effect of composting processes of raw municipal sewage sludge on mycotoxins and total heavy metals contents, the mycotoxins heavy metals were determined and the results showed that the mycotoxins contents of viomelline to be 4,903.78 ppb, aflatoxins 20.90, and moniliformin 2,688 in rice straw, while in clay soil and aflatoxins the value was 2.293 ppb. The results exhibited that the conventional process can reduce MSS mycotoxins content by 36.23%. Our findings were identical with those of^62^ who mentioned that the reduction of mycotoxins is generally correlated with the degree of heat applied in the process; however, heat energy may not cause complete elimination of mycotoxins during the incubation period. Most mycotoxins can be reduced at temperatures above 150 °C. Our results can relatively coincide with^5^ and^30^ in their target to recycle the bio-MSS waste into a useful and beneficial product for soil. All resulting heavy metal elements concentration, except Al, were below their normal range in soil and plant according to their permissible limits recommended by WHO, FAO, Dutch, Austrian, Russian, and European Union standards^63^. So, this conventional composting process was safer for soil addition. Our findings were in agreement with those of^64^ who reported that composting process serve to eliminate heavy metals and phytotoxic substance.

The treatment of raw primary treated MSS or bio-solids with probiotic (*LAB* mixture) and/or fungus was effective in reducing both of mycotoxins and heavy metals, but, unfortunately, the concentration of some heavy metals after the biotic treatments are still higher than the allowable limits in air, soil, and plants, which may be the case due to the short period of treatment. Our results showed that the composting process of MSS was vital to reducing biotic and abiotic pollutants. Furthermore, the biotic composition process was the best treatment in removing both mycotoxins and heavy metals from municipal sewage sludge. Although the levels of heavy metals vary greatly in their concentrations among different Mediterranean soils, our obtained HMs concentration in the present study are below the mentioned concentrations in different studies. The resulting MSS compost became safer for soil. Our study does not encourage the usage of raw municipal sewage sludge as woody trees fertilizer but tries to solve its harmful usage effects to the soil. According to European Union legislation on sewage sludge management, the sewage sludge can be added to the soil for a maximum of three years only, but our biotical-treated compost can be used to save soil amendments with stabilization of organic substrates. According to^65^, the composting of MSS is recommended as a way of transforming a less stabilized waste into a beneficial material. The biotic composting treatment with *S. indica* or the *LAB* mixture is recommended; they are very cheap and economically available composting processes, especially in developed countries. Composting is considered to be a vital technology to recycle biodegradable waste while generating a useful product.
